# NLRP3/caspase-1/GSDMD–mediated pyroptosis exerts a crucial role in astrocyte pathological injury in mouse model of depression

**DOI:** 10.1172/jci.insight.146852

**Published:** 2021-12-08

**Authors:** Shanshan Li, Yiming Sun, Mengmeng Song, Yuting Song, Yinquan Fang, Qingyu Zhang, Xueting Li, Nanshan Song, Jianhua Ding, Ming Lu, Gang Hu

**Affiliations:** 1 Department of Pharmacology, Nanjing University of Chinese Medicine, Nanjing, Jiangsu, China.; 2 Jiangsu Key Laboratory of Neurodegeneration, Department of Pharmacology, and; 3 Neuroprotective Drug Discovery Key Laboratory, Department of Pharmacology, Nanjing Medical University, Nanjing, Jiangsu, China.

**Keywords:** Inflammation, Cytokines, Depression, Psychiatric diseases

## Abstract

Emerging evidence suggests that astrocyte loss is one of the most important pathological features in the hippocampus of patients with major depressive disorder (MDD) and depressive mice. Pyroptosis is a recently discovered form of programmed cell death depending on Caspase–gasdermin D (Casp-GSDMD), which is involved in multiple neuropsychiatric diseases. However, the involvement of pyroptosis in the onset of MDD and glial pathological injury remains obscure. Here, we observed that depressive mice showed astrocytic pyroptosis, which was responsible for astrocyte loss, and selective serotonin reuptake inhibitor (SSRI) treatment could attenuate the pyroptosis induced by the chronic mild stress (CMS) model. Genetic KO of GSDMD, Casp-1, and astrocytic NOD-like receptor protein 3 (NLRP3) inflammasome in mice alleviated depression-like behaviors and inhibited the pyroptosis-associated protein expression. In contrast, overexpression of astrocytic GSDMD–N-terminal domain (GSDMD-N) in the hippocampus could abolish the improvement of behavioral alterations in GSDMD-deficient mice. This work illustrates that targeting the NLRP3/Casp-1/GSDMD–mediated pyroptosis may provide potential therapeutic benefits to stress-related astrocyte loss in the pathogenesis of depression.

## Introduction

Major depressive disorder (MDD) is a major health problem affecting about 300 million people globally (1, 2). The onset of depression is closely associated with a combination of physical changes and physiological stress, contributing to psychosocial dysfunction and decreased quality of life (3). Although much progress has been made in antidepressant treatment, nearly 80% of patients experience at least one recurrence in their lifetime (4). At present, the exact pathophysiological mechanism of depression is still elusive, various hypotheses have been proposed to explain this disease, including monoamine neurotransmitter disturbance, hypothalamic/pituitary/adrenal (HPA) axis disorder, neurotrophic factor deficiency, and immune dysfunction (5, 6). Increasing evidence shows that classic antidepressants targeting serotonergic neurons may be ineffective in a considerable part of patients (7). Thus, the specific therapeutic strategies are required to be developed for the treatment of MDD.

Accumulating studies have shown that astrocytes are highly correlated to the pathophysiological process of depression (8). Particularly, the number of glial fibrillary acidic protein^+^ (GFAP^+^) astrocytes are decreased in the hippocampus and medial prefrontal cortex (mPFC) of patients with MDD (9) and a chronic mild stress–induced (CMS-induced) rodent model (10). Effective treatments in MDD can enhance the proliferation and gene expression of astrocytes (11, 12), while the gliotoxin-induced reduction in the number of astrocytes in the mPFC led to depression-like phenotypes of rats (13). In addition, the results of autopsy samples show that the density of astrocytes and GFAP immunoreactivity in the dorsolateral prefrontal cortex have changed and are dependent on age. Compared with the age-matched control group, the cell density and immunoreactivity of GFAP are lower in young MDD subjects (14, 15). However, the cause of astrocytic loss in the pathogenesis of depression remains unknown.

Pyroptosis is a form of cell death mediated by gasdermin (GSDM) family protein (e.g., GSDMD and GSDME), and it is characterized by activation of the NOD-like receptor protein 3 (NLRP3) inflammasome, the formation of cell membrane pore, and release of IL-1β and IL-18 (16, 17). The GSDMD–N-terminal domain (GSDMD-N) is generated from GSDMD protein cleaved by proinflammatory Caspases (Casps) through classical and nonclassical inflammasome signaling pathways, and then rapidly inserts into the plasma membrane to form an active pore (18, 19). Therefore, GSDMD-N acts as the ultimate executor of pyroptosis. The NLRP3 inflammasome is one of the largest and most representative inflammasomes to date, contains NLRP3, apoptosis-associated speck-like protein (ASC), and pro–Casp-1 protein (20). NLRP3 has high expression levels in astrocytes, the activation of which plays a predominant role in pyroptosis (21). The activation of pyroptosis has been reported in various neurodegenerative diseases (NDD), such as multiple sclerosis (MS) (22), Parkinson’s disease (PD) (23), and Alzheimer’s disease (AD) (24). A recent study reveals that suppression of NLRP3/Casp-1–dependent pyroptosis provides a defense mechanism against α-synuclein (α-syn) aggregate–induced dopaminergic neuron loss, suggesting that pyroptosis participates in the dopaminergic neurodegeneration (25). However, the link between pyroptosis and the loss of astrocytes in the hippocampus and mPFC during the pathogenesis of depression is still obscure.

Given the astrocytic loss in depression and the correlation between pyroptosis and nervous system diseases, it is tempting to speculate that the NLRP3/Casp-1/GSDMD–dependent pyroptotic process contributes to the astrocytic loss in the context of depression. In the present study, we tested this hypothesis by detecting the depression-like behaviors of WT mice and evaluating whether pyroptosis in the hippocampus is associated with the decreased number of astrocytes in the CMS mouse model. We revealed that depression was accompanied by the increased levels of pyroptosis-associated markers in the mouse hippocampus and that selective serotonin reuptake inhibitor (SSRI) treatment could attenuate the pyroptosis induced by the CMS model. Besides, astrocyte-specific NLRP3 conditional KO (NLRP3-cKO), Casp-1–KO (Casp-1^–/–^), and GSDMD-KO (GSDMD^–/–^) mice were manipulated to restrain the NLRP3/Casp-1/GSDMD signal transduction pathway in mouse astrocytes, and the adenoviral vector was used to make astrocytic GSDMD-N overexpressed to allow for the evaluation of its critical role in the pathogenesis of depression and the clarification of the possible molecular mechanism for astrocytic loss in the development of depression.

## Results

### Astrocytic pyroptosis is induced in the hippocampus of mice after CMS stimulation for 4 weeks.

**Figure 1:**
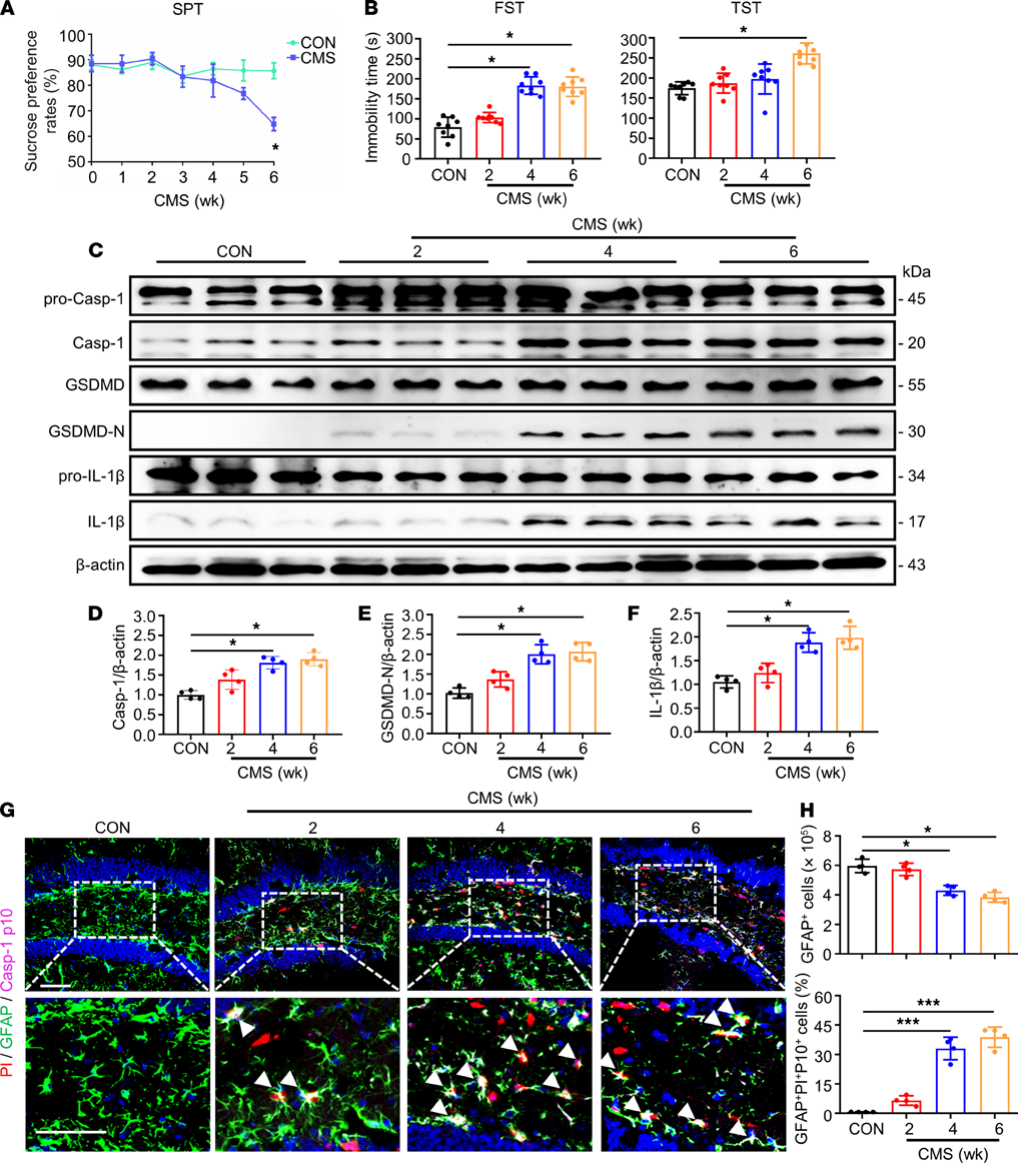
The pyroptosis of astrocytes was induced in the hippocampus of mice after CMS stimulation for 2 weeks. WT mice were performed to CMS stimulation for 2, 4, and 6 weeks. Behavioral tests were then conducted. (**A**) The SPT was recorded every week for 6 weeks. (**B**) The immobility time in the FST and TST was implemented after the last administration. *n* = 8 mice per group. (**C**–**F**) Immunoblotting was used to analyze the expression of pro–Casp-1, Casp-1, GSDMD, GSDMD-N, pro–IL-1β, and IL-1β in the hippocampus. Densitometric analysis of Casp-1 (**D**), GSDMD-N (**E**), and IL-1β (**F**); *n* = 4 mice per group. (**G**) Representative high-magnification images showing the colocalization of GFAP (green), Casp-1 p10 (magenta), and PI (red) in a portion of the ipsilateral DG hippocampal region from 1 animal injected with vehicle or 1 μL of PI following CMS stimulation. White arrowheads represent that example of PI^+^/GFAP^+^/Casp-1 p10^+^ cells. (**H**) Densitometric analysis of the numbers of GFAP^+^ cells and percentage of PI^+^/GFAP^+^/Casp-1 p10^+^ cells in the DG area of hippocampus. Scale bar: 50 μm. *n* = 4 mice per group. Values were represented as mean ± SEM. Data were analyzed using Student’s *t* test. *P < 0.05, ***P <0.001.

To investigate whether pyroptosis was induced by CMS stimulation and its dynamic characteristics in the mouse brain, 3 time points — 2-week, 4-week, and 6-week periods — were set in the entire CMS model. The levels of pyroptosis-associated markers were measured by Western blotting and Tyramide signal amplification–coupled (TSA) multiplex fluorescence staining. Depression-like behaviors were tested by forced swim test (FST), tail suspension test (TST), and sucrose preference test (SPT) after CMS stimulation for 2, 4, and 6 weeks. In the SPT, CMS mice manifested a significant reduction in sucrose consumption compared with that in the control group observed at the end of week 6 (Figure 1A). In the FST, CMS mice showed a noticeable increase in the immobility time in weeks 4 and 6 compared with times in the controls (Figure 1B). In the TST, CMS mice exhibited a sharp increase in the immobility time in week 6 compared with that in the controls (Figure 1B). These results indicated that CMS induced depression-like behaviors of mice at the end of week 6. Next, we tested the expression of pyroptosis-associated protein levels in the hippocampus of mice. Compared with control groups, the expression levels of cleaved Casp-1, GSDMD-N, and mature IL-1β were enhanced in the hippocampus after CMS stimulation for 4 and 6 weeks (Figure 1, C–F). Moreover, the decreased number of GFAP^+^ cells and increased multilabeled fluorescence intensity of propidium iodide (PI), GFAP, and cleaved Casp-1 (p10) were observed in CMS mice after stimulation for 4 and 6 weeks (Figure 1, G and H). These results suggest that loss and pyroptosis of astrocytes were induced in the hippocampus of mice during the pathological process of depression.

Next, we detected the distribution of pyroptosis in different brain regions after the injection of vehicle or 1 μL of PI into the lateral ventricle following 6-week CMS stimulation. As shown in Supplemental Figure 1 and Supplemental Figure 2, A and B (supplemental material available online with this article; https://doi.org/10.1172/jci.insight.146852DS1), the decreased number of GFAP^+^ cells and increased fluorescence colocalization of GFAP^+^PI^+^Casp-1 p10^+^ cells were clearly observed only in the hippocampus, not in the cortex, striatum, or hypothalamus, indicating that pyroptosis of astrocytes predominantly appeared in the hippocampus of CMS mice.

Besides pyroptosis, many other cell death forms have been identified, such as apoptosis and necroptosis (26). To investigate the proportion of pyroptosis among cell death forms in depression, we evaluated the expression of apoptosis and necroptosis in astrocytes from the hippocampus of mice. Confocal fluorescence image results show that, compared with the control groups, the colocalization of PI^+^ and Casp-1 p10^+^ cells in astrocytes from CMS groups was 39.84% (Supplemental Figure 2, A and B). By contrast, we found that the percentage of Casp-3^+^ and pMLKL^+^ cells in astrocytes from CMS groups was 14.87% (Supplemental Figure 2, C and D) and 5.02% (Supplemental Figure 2, E and F), respectively, implying that pyroptosis is the most predominant form of cell death of astrocytes in the CMS model of depression.

Moreover, to verify whether pyroptosis appeared in microglia and neuron from the hippocampus of CMS mice, we also assessed the colabeled immunofluorescence levels of Iba-1 or NeuN with PI and Casp-1 p10, respectively. Results showed that double-labeled immunofluorescence levels of Casp-1 p10 and PI were not detected in Iba-1^+^ or NeuN^+^ cells (Supplemental Figure 3). These results further reveal that hippocampal pyroptosis only occurred in astrocytes other than microglia or neurons after 6-week CMS stimulation.

### SSRIs treatment alleviated pyroptosis of astrocytes in the hippocampus of CMS mice.

**Figure 2:**
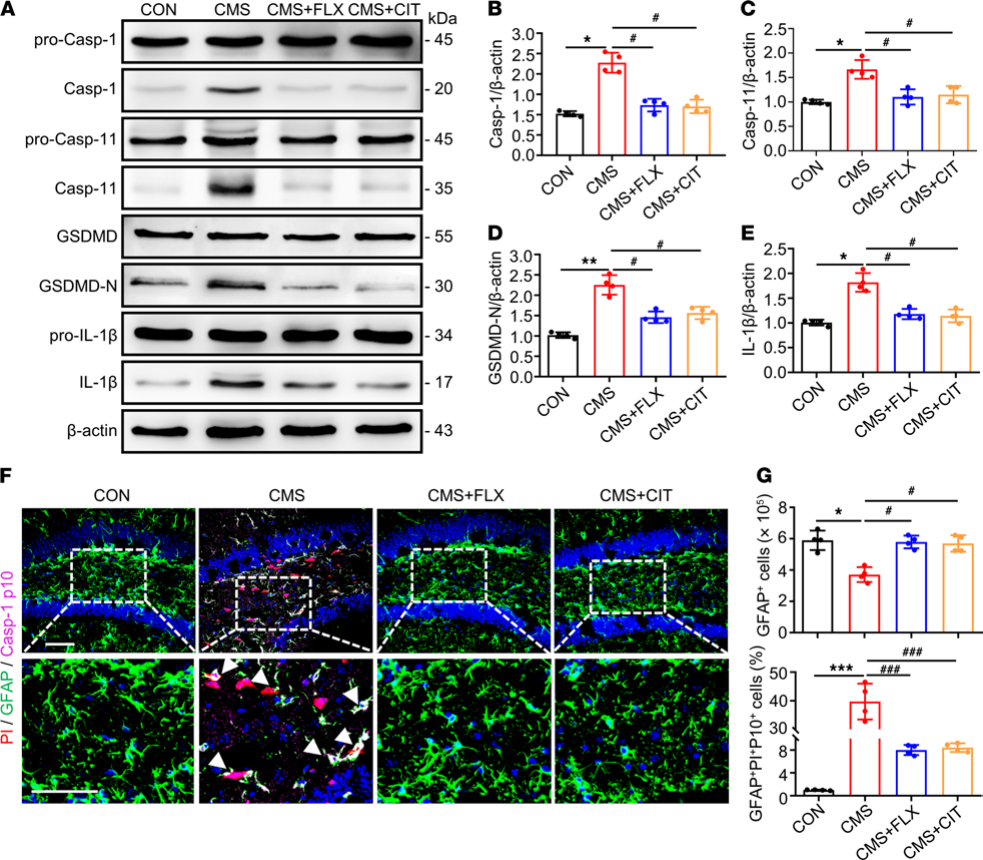
SSRIs alleviated levels of astrocytic pyroptosis in the hippocampus of CMS mice. (**A**) Immunoblotting was used to analyze the expression of pro–Casp-1, Casp-1, pro–Casp-11, Casp-11, GSDMD, GSDMD-N, pro–IL-1β, and IL-1β from mice hippocampus. (**B**–**E**) Densitometric analysis of Casp-1 (**B**), Casp-11 (**C**), GSDMD-N (**D**), and IL-1β (**E**). *n* = 4 mice per group. (**F**) Representative high-magnification images showing the localization of GFAP (green), Casp-1 p10 (magenta), and PI (red) in a portion of the ipsilateral DG hippocampal region from 1 animal injected with vehicle or 1 μL of PI following CMS stimulation. White arrowheads represent that example of PI^+^/GFAP^+^/Casp-1 p10^+^ cells. (**G**) Densitometric analysis of the numbers of GFAP^+^ cells and percentage of PI^+^/GFAP^+^/Casp-1 p10^+^ cells in the DG area of hippocampus. Scale bar: 50 μm. *n* = 4 mice per group. Values were represented as mean ± SEM. Data were analyzed using 1-way ANOVA. They were then combined with unpaired *t* test to assess the differences between groups. *P < 0.05, **P <0.01, ***P <0.01 versus CON group; ^#^*P* < 0.05, ^###^*P* < 0.001 versus CMS group.

SSRIs are the most used antidepressants in clinical practice, and fluoxetine (FLX) and citalopram (CIT) are representative drugs among them (27). Beyond their influence on the monoaminergic neurotransmission, FLX and CIT also exhibited the potential impacts on rescuing depression-like behaviors via improving astrocyte loss (28, 29). Thus, we studied the association between antidepressant actions of FLX and CIT with the pyroptosis in astrocytes. As shown in Figure 2, the activation of pyroptosis-associated proteins, including Casp-1/11, GSDMD-N, and IL-1β, as well as immunofluorescence levels of Casp-1 p10^+^, PI^+^, and GFAP^+^ cells were increased in hippocampal tissues of CMS mice; this activation could be reversed by the treatment of FLX or CIT. These results illustrate that the SSRIs could alleviate astrocytic pyroptosis in the hippocampus of mice. Moreover, we observed that numbers of GFAP^+^ cells and protein levels of GFAP were drastically reduced in the hippocampus of CMS mice, and this could be alleviated with the treatment of FLX or CIT (Supplemental Figure 4 and Figure 2, F and G), suggesting that SSRIs could alleviate both pyroptosis and loss of astrocytes in the hippocampus of CMS mice.

### GSDMD participates in the pyroptosis of astrocytes and astrocytic loss in CMS mice.

**Figure 3:**
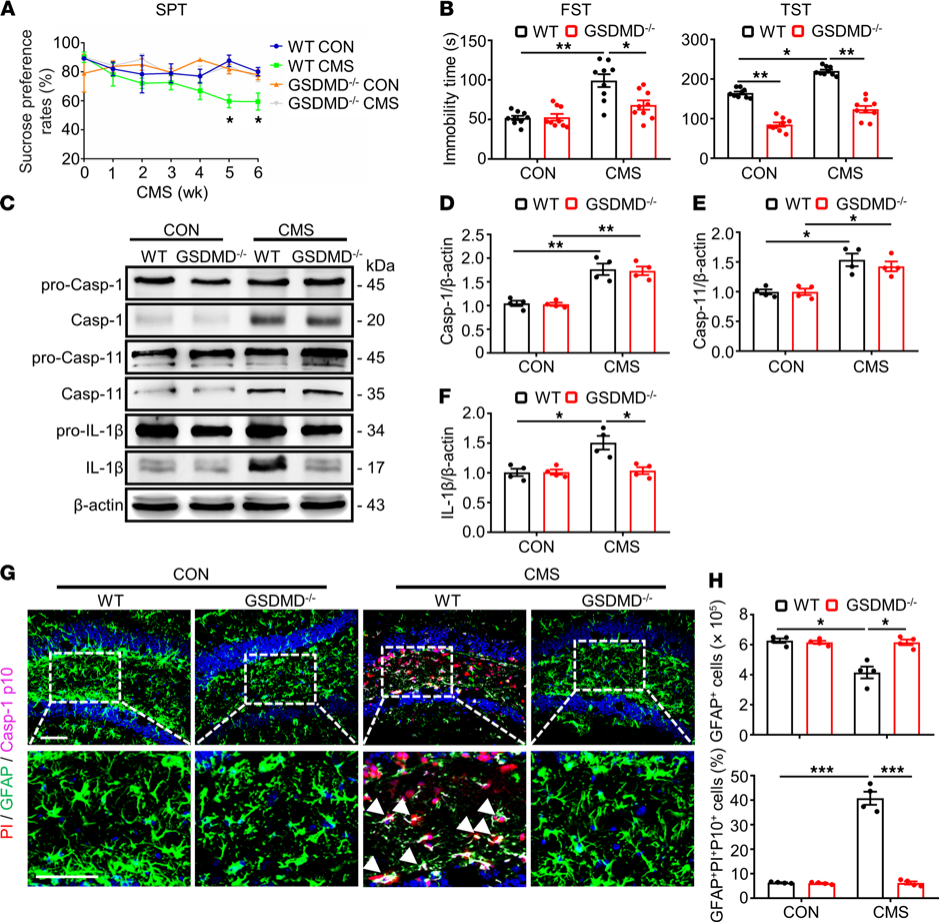
GSDMD KO attenuated the pyroptosis of astrocytes in the hippocampus of CMS mice. (**A**) The SPT of mice was recorded weekly during the 6-week CMS period. (**B**) The immobility time in the FST and TST was conducted after CMS stimulation; *n* = 9 mice per group. Values were represented as mean ± SEM. Data were analyzed using 2-way ANOVA and were then combined with Tukey to assess the differences between groups. (**C**–**F**) Immunoblotting was used to analyze the expression of pro–Casp-1, Casp-1, pro–Casp-11, Casp-11, pro–IL-1β, and IL-1β from mouse hippocampus homogenate. Densitometric analysis of Casp-1 (**D**), Casp-11 (**E**), and IL-1β (**F**); *n* = 4 mice per group. (**G**) GFAP-labeled (green), Casp-1 p10–labeled (magenta), and PI-labeled (red) cells in a portion of the ipsilateral DG hippocampal region from 1 animal injected with vehicle or 1 μL of PI following CMS stimulation by TSA coupled multiplex fluorescence staining. White arrowheads represent that example of PI^+^/GFAP^+^/Casp-1 p10^+^ cells. (**H**) Densitometric analysis of numbers of GFAP^+^ and percentage of GFAP^+^, Casp-1 p10^+^, and PI^+^ cells in the DG region of hippocampus. Scale bar: 50 μm. *n* = 4 mice per group. Values were represented as mean ± SEM. Data were analyzed using 2-way ANOVA and were then combined with Tukey to assess the differences between groups. *P < 0.05, **P < 0.01, ***P < 0.001.

Studies have been reported that GSDMD was a downstream executor in mediating the process of pyroptosis (30). We observed that the CMS model increased GSDMD-N protein expression in the hippocampus of mice from the above results (Figure 1, C and E, and Figure 2, A and D). Therefore, we first discussed the effects of GSDMD on the pyroptosis of astrocytes. GSDMD^–/–^ mice and their littermate control WT mice were stimulated by the CMS model. Before the CMS stimulation, we used Western blotting to validate the GSDMD KO efficiency by detecting the hippocampal protein levels in mice (Supplemental Figure 5). Then, the depression-like behaviors were determined in GSDMD^–/–^ mice and their littermate control WT mice. Sucrose preference was significantly reduced in WT mice after CMS treatment for 6 weeks, and this preference could be remarkably restored by GSDMD KO (Figure 3A). Similarly, compared with control animals, the immobility time in the FST and TST, induced by CMS were reversed by GSDMD KO (Figure 3B). These results implied that GSDMD participated in CMS-induced depression–like behaviors in mice.

Then, the effects of GSDMD on the pyroptosis of astrocytes induced by CMS model were measured using immunoblotting and immunostaining. Compared with control groups, the protein level of mature IL-1β induced by CMS were inhibited by GSDMD KO (Figure 3, C–F). Moreover, GSDMD KO restored the decreased number of GFAP^+^ cells and attenuated the strengthened multilabeled fluorescence intensity of PI, GFAP, and Casp-1 p10 induced by the CMS model (Figure 3, G and H). These results suggest that GSDMD may be the key factor in depression-like behaviors and the pyroptosis of astrocytes in the CMS model.

**Figure 4:**
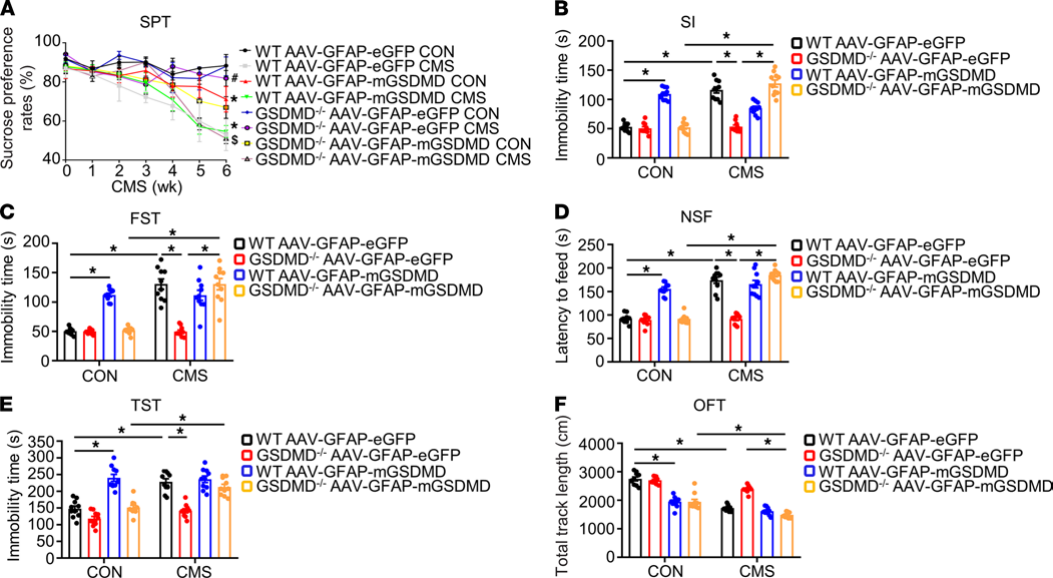
Overexpression of GSDMD-N in astrocytes reversed the amelioration of depression-like behaviors in GSDMD^–/–^ mice. (**A**) The SPT of mice was recorded weekly during the 6-week CMS period. Values were represented as mean ± SEM. *P < 0.05 versus respective CON WT mice injected with AAV-GFAP-EGFP group; ^#^*P* < 0.05 versus respective CMS WT mice injected with AAV-GFAP-EGFP group; ^$^*P* < 0.05 versus respective GSDMD^–/–^ mice injected with AAV-GFAP-mGSDMD. (**B**–**F**) latency to suiffing in the SI (**B**), latency to feed in the NSF (**D**), the immobility time in the FST (**C**) and TST (**E**), and total track length in the OFT (**F**) were conducted after CMS stimulation; *n* = 10–11 mice per group. Values were represented as mean ± SEM. Data were analyzed using repeated-measures ANOVA and were then combined with post hoc Tukey-Kramer test to assess the differences between groups. *P < 0.05.

To further study the role of GSDMD in the pyroptosis of astrocytes in depression, an adeno-associated virus (AAV) vector (serotype 9) with an EGFP that selectively overexpresses GSDMD-N in astrocytes (AAV-GFAP-mGSDMD-EGFP) was injected into the hippocampal region of GSDMD^–/–^ mice and their control WT mice (Supplemental Figure 6A). Overexpressing GSDMD-N in astrocytes showed drastic overexpression efficacy 4 weeks after AAV-GSDMD infusion (Supplemental Figure 6, B and C). Then, the mice were subjected to CMS model for 6 weeks (Supplemental Figure 6D). As shown in Figure 4, CMS stimulation markedly strengthened the immobility both in FST and TST and reduced the sucrose preference in SPT of WT mice treated with AAV-GFAP-EGFP; these changes were reversed in GSDMD^–/–^ mice treated with AAV-GFAP-EGFP (Figure 4, A, C, and E). Furthermore, CMS stimulation exposed that WT mice treated with AAV-GFAP-mGSDMD-EGFP had markedly strengthened the latency to feed and to partake in social interaction (SI) and novelty-suppressed feeding (NSF) compared with control WT mice treated with AAV-GFAP-EGFP, and this latency could be reversed in GSDMD^–/–^ mice treated with AAV-GFAP-EGFP (Figure 4, B and D). The result of open-field test (OFT) showed that GSDMD KO markedly restored the total moving distance of WT mice treated with AAV-GFAP-EGFP after CMS stimulation (Figure 4F). By contrast, CMS stimulation exposed that GSDMD^–/–^ mice treated with AAV-GFAP-mGSDMD-EGFP could significantly abolish the improved tendency of depression-like behaviors, compared to those of GSDMD^–/–^ mice treated with AAV-GFAP-EGFP (Figure 4). The above results demonstrate that GSDMD was responsible for the depression-like behaviors, the loss of astrocytes, and the pyroptosis of astrocytes in the CMS model.

### Casp-1 is required for the GSDMD-N induction and the pyroptosis of astrocytes in response to CMS stimulation.

**Figure 5:**
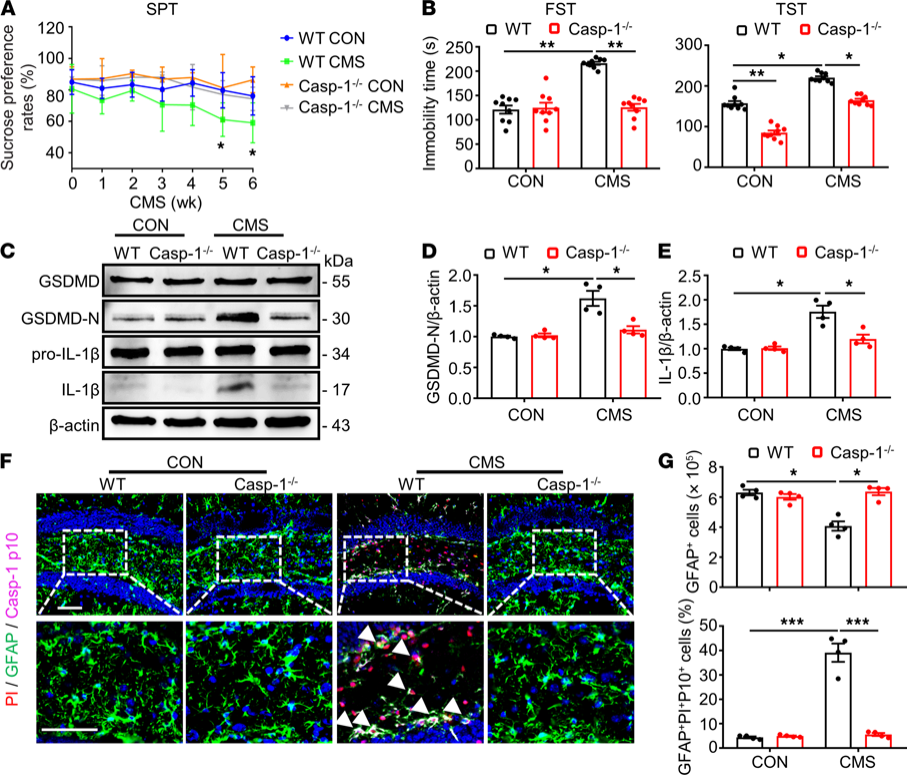
Casp-1 KO improved the proptosis of astrocytes in the hippocampus of CMS mice. (**A**) The SPT of mice was recorded weekly during the 6-week CMS period. (**B**) The immobility time in the FST and TST were implemented at 30 minutes after the last administration. *n* = 9 mice per group. (**C**–**E**) Immunoblotting was used to analyze the expression of GSDMD, GSDMD-N, pro–IL-1β, and IL-1β from mouse hippocampus homogenate. Densitometric analysis of GSDMD-N (**D**) and IL-1β (**E**). *n* = 4 mice per group. (**F**) GFAP-labeled(green), Casp-1 p10–labeled (magenta), and PI-labeled (red) cells in a portion of the ipsilateral DG hippocampal region from 1 animal injected with vehicle or 1 μL of PI following CMS stimuli by TSA coupled multiplex fluorescence staining. White arrowheads represent that example of PI^+^/GFAP^+^/Casp-1 p10^+^ cells. (**G**) Densitometric analysis of numbers of GFAP^+^ and percentage of GFAP^+^/Casp-1 p10^+^/PI^+^ cells in the DG region of hippocampus. Scale bar: 50 μm. *n* = 4 mice per group. Values were represented as mean ± SEM. Data were analyzed using 2-way ANOVA and were then combined with Tukey to assess the differences between groups. *P < 0.05, **P < 0.01, ***P < 0.001.

Previous studies have indicated that Casp-1 is responsible for GSDMD induction in the process of pyroptosis under different stimulation and diverse cellular contexts (31, 32). Besides, obvious results showed that the protein level of Casp-1 was increased in a sustained manner in the pathogenesis of CMS stimulation (Figure 1, C and D). Thus, we further studied the potential role of Casp-1 in astrocyte pyroptosis in the hippocampus of CMS mice. CMS model was performed on Casp-1^–/–^ mice and their littermate WT mice.. Sucrose preference was significantly reduced in WT mice after CMS treatment for 6 weeks, which could be remarkably restored by Casp-1 KO (Figure 5A). Equally, the immobility time was drastically prolonged in the FST and TST after CMS stimulation, which could be reversed by Casp-1 KO (Figure 5B).

Then the effects of Casp-1 on the CMS model induced pyroptosis of astrocyte were measured using immunoblotting and immunostaining. Compared with the controls, the protein levels of GSDMD-N and IL-1β induced by CMS were inhibited by Casp-1 KO (Figure 5, C–E). Moreover, the decreased number of GFAP^+^ cells and the strengthened multilabeled fluorescence intensity of PI, GFAP, and Casp-1 p10 induced by the CMS model were significantly recovered by Casp-1 KO (Figure 5, F and G). These results suggest that GSDMD-N induction, the loss of astrocytes, and the pyroptosis of astrocytes in the CMS model were mediated by Casp-1. In conclusion, the above results showed that the Casp-1/GSDMD pathway is vital in CMS-induced astrocytic loss and pyroptosis.

### Astrocytic NLRP3 inflammasome is essential for CMS-induced GSDMD-N expression and pyroptosis of astrocytes in the hippocampus of CMS mice.

**Figure 6:**
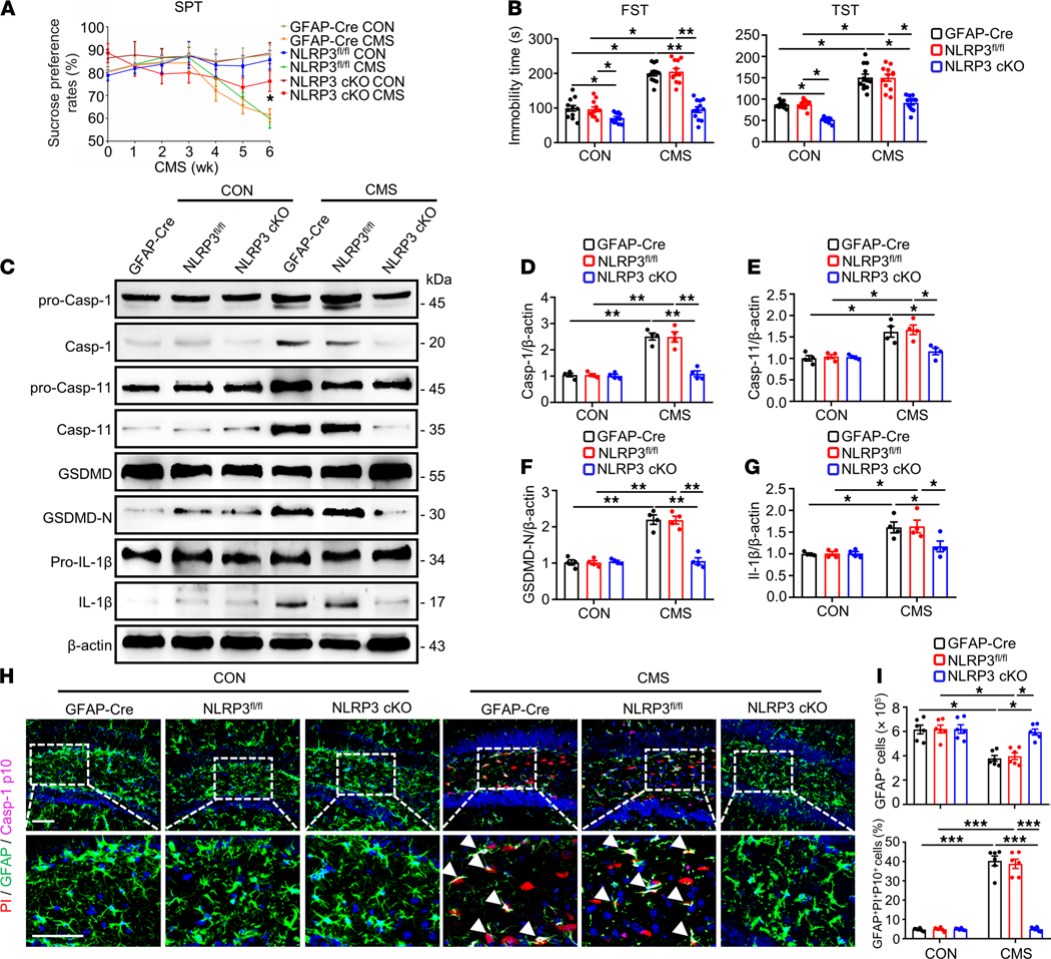
Astrocytic NLRP3 KO improved the proptosis of astrocyte in the hippocampus of CMS mice. (**A**) The SPT of mice was recorded weekly during the 6-week CMS period. (**B**) The immobility time in the FST and TST were tested at 30 minutes apart. *n* = 12 mice per group. (**C**–**G**) Immunoblotting was used to analyze the expression of pro–Casp-1, Casp-1, pro–Casp-11, Casp-11, GSDMD, GSDMD-N, pro–IL-1β, and IL-1β in the hippocampus of mice. Densitometric analysis of Casp-1 (**D**), Casp-11 (**E**), GSDMD-N (**F**), and IL-1β (**G**). *n* = 4 mice per group. (**H**) Representative images showed the colocalization of GFAP (green), Casp-1 p10 (magenta), and PI (red) in a portion of the ipsilateral DG hippocampal region from 1 animal injected with vehicle or 1 μL of PI following CMS stimulation. White arrowheads represent that example of PI^+^/GFAP^+^/Casp-1 p10^+^ cells. (**I**) Densitometric analysis of numbers of GFAP^+^ and percentage of GFAP^+^/Casp-1 p10/PI^+^ cells in the DG region of hippocampus. Scale bar: 50 μm. *n* = 6 mice per group. Values were represented as mean ± SEM. Data were analyzed using 2-way ANOVA and were then combined with Tukey to assess the differences between groups. *P < 0.05, **P < 0.01, ***P < 0.001.

The induction of pyroptosis-related protein GSDMD and proinflammatory cytokine IL-1β is reported to be regulated by the activation of NLRP3 inflammasome (33). In addition, Casp-1 was reported to mediate the effects of NLRP3 inflammasome in secreting of inflammatory cytokines and inducing inflammatory cell death, or pyroptosis (34). Accordingly, we next evaluated whether NLRP3 inflammasome related to CMS induced pyroptosis of astrocytes. Astrocyte-specific NLRP3 conditional KO mice (NLRP3 cKO) and their littermates NLRP3^fl/fl^ mice and GFAP-Cre mice were used to prepare the CMS model and depression-like behaviors and pyroptosis of astrocytes were determined (Supplemental Figure 7A). The genotyping of NLRP3-cKO mice was validated for DNA levels by PCR (Supplemental Figure 7, B and C). We observed that the downregulated sucrose preference induced by the CMS model was improved in NLRP3-cKO mice (Figure 6A). FST and TST results show that the prolonged immobility time in the CMS model was markedly reversed in NLRP3-cKO mice (Figure 6B). These findings imply that astrocytic NLRP3 inflammasome is associated with CMS-induced depression–like behaviors in mice.

To elucidate the potential role of astrocytic NLRP3 inflammasome involved in CMS model–induced astrocyte pyroptosis, we tested pyroptosis-associated protein levels in the hippocampus of mice. Compared with control groups, the CMS model induced a significant increase in the expression of hippocampal Casp-1/11, GSDMD-N, and IL-1β, while astrocytic NLRP3 KO downregulated these proteins levels (Figure 6, C–G). Moreover, astrocytic NLRP3 KO restored the loss of GFAP^+^ cells and an increase in the multilabeled fluorescence intensity of PI, GFAP, and Casp-1 p10 in the mouse hippocampus induced by CMS model (Figure 6, H and I). These results suggest that astrocytic NLRP3 inflammasome plays a critical role in mediating loss and pyroptosis of astrocytes and in GSDMD-N induction in CMS mice. Taken together, our data therefore show that the NLRP3/Casp-1/GSDMD pathway is critical for CMS-induced astrocytic loss and pyroptosis.

## Discussion

In this study, we suggest a potentially new form of astrocytes loss to respond to CMS stimulation partly via the NLRP3/Casp-1/GSDMD–mediated pyroptotic pathway. We reveal that depression was accompanied by increased levels of pyroptosis-associated markers in the hippocampus. Administration with SSRIs significantly could attenuate the pyroptosis in astrocytes induced by CMS model. Mechanistically, GSDMD KO protected against CMS stimulation and decreased the levels of astrocytic pyroptosis and astrocytic loss, whereas hippocampal GSDMD-N overexpression in astrocytes abolished the improvement of depression-like phenotypes in GSDMD^–/–^ mice. Furthermore, astrocyte-specific NLRP3 KO and Casp-1 KO both protected against CMS stimulation and decreased the levels of astrocytic pyroptosis and astrocytic loss similarly to the results obtained from knocking out the GSDMD gene in mice.

Depression-induced astrocytic loss has been observed in the hippocampus of rodents that present with depression and autopsy specimens of patients with MDD (35), but the cause of astrocytic loss remains unknown. Pyroptosis is a potentially new form of programmed cell death that is different from apoptosis and ferroptosis (36, 37). Recent studies have shown that blockade of the NLRP3/Casp-1/IL-1β pathway may alleviate the loss of dopaminergic neurons induced from the α-syn aggregates, indicating that pyroptosis may play a vital role in the death process of dopaminergic neurons (38). Also, the blockade of the Casp-1/IL-1β pathway may prevent Müller cell loss induced by diabetes, indicating that the pyroptotic process may be responsible for Müller cell death (39). Based on the above studies, we speculated that astrocytic loss, induced in the hippocampus of mice by CMS stimulation, was partly due to the pyroptosis of astrocytes. Since the pathogenesis of depression was chronic and complicated, we first investigated the dynamic characteristics of pyroptosis in the pathogenesis of depression. Our results show that astrocytic pyroptosis was induced after CMS stimulation from 4 weeks to 6 weeks, indicating that the pyroptosis of astrocytes existed in the hippocampus during the pathogenesis of the CMS model and might be the cause for the depression-like phenotype of CMS mice. A recent study found that neuronal pyroptosis increasingly occurred during the whole pathological process of spinal cord injury (SCI) and led to the neurological dysfunction in this disease (40). This finding further proved that pyroptosis played an important role in pathological processes of mental and neurological disorders.

Our fluorescence analysis further revealed that the hippocampus was the major brain region where loss and pyroptosis of astrocytes occurred in depression. The former had a confirmed histological diagnosis both in patients with MDD and depressive rodent models (3, 35). Then, we found that — in contrast to apoptosis and necrosis, which have been reported to be related to depression — pyroptosis was the dominant form in astrocytic death from the CMS mice hippocampal region. Our fluorescence results further validate that hippocampal pyroptosis existed only in astrocytes other than other cell types (i.e., microglia and neurons) in CMS mice. Taken together, our results first reveal that hippocampus was the major brain region where loss and pyroptosis of astrocytes occurred in depression. Next, the fluorescence and Western blotting analysis demonstrate that the CMS model induced astrocytic loss and increased the levels of pyroptosis-associated markers in the hippocampus of mice as expected, and this could be reversed by the treatment of SSRIs. FLX or CIT treatment has been reported to fully abolish the depression-like behaviors of rodents in both chronic social defeat stress (CSDS) and the CMS models of depression (41, 42). Collectively, these findings imply the idea that, apart from a recognized monoaminergic system (43), astrocytic pyroptosis could be a potential antidepressant target of SSRIs.

In basal conditions, GSDMD is located only in the cytosol and can be cleaved by Casps to produce GSDMD-N, which forms a pore-like structure in the lipid membrane and, thus, acts as the direct effector of pyroptosis (44, 45). In addition, GSDMD-N alone induces pyroptosis when ectopic expression occurs (18). Up to now, all studies have concluded that the pore-formation characteristics of GSDMD-N are the driving factors of pyroptosis, given the well-known role of GSDMD-N as a downstream molecule in pyroptosis and that the mechanism of GSDMD in depression has not been studied. Positive and negative regulation of GSDMD via virally mediated overexpression of astrocytic GSDMD-N and genetic GSDMD KO strongly support the possibility that GSDMD could be a therapeutic target in the astrocytic pyroptosis in depression.

Next, we examined the mechanism by which CMS stimulation–induced astrocytic pyroptosis participated in the loss of hippocampal astrocytes, given that CMS stimulation induced the hippocampal Casp-1 activation and Casp-1 was reported to be the GSDMD upstream cascade pathway member to initiate the pyroptosis (46, 47). Additionally, reports proved that the activation of the Casp-1/IL-β signaling pathway could induce anxiety- and depression-like behaviors of mice (48). It would be very interesting to see whether Casp-1 participated in astrocytic loss and astrocytic pyroptosis in the hippocampus of CMS mice. Using Casp-1^–/–^ mice, it was convincingly demonstrated that Casp-1 is indeed an important mediator for the hippocampal Casp-1/GSDMD–mediated pyroptotic process in the astrocytic loss.

Furthermore, it has been reported that the assembly of a multiprotein, defined as inflammasome or pyroptosome, provided a platform to induce the activation of Casp-1 (47). The activation of NLRP3 inflammasome gives rise to the maturation of Casp-1, which secretes the downstream cytokines IL-1β and IL-18, making them biologically active and inducing pyroptosis (49). Besides, the activation of NLRP3 inflammasome assembly was reported to be capable to induce the depression-like behaviors of mice by LPS injection or chronic unpredictable mild stress (CUMS) stimulation (50, 51). The astrocytic NLRP3 KO results reported here suggest that astrocytic NLRP3 inflammasome had a significant effect on the astrocytic pyroptosis via the downstream Casp-1/GSDMD pathway in the pathophysiology of depression. The results were fully demonstrated by behavioral measures and the levels of astrocytic pyroptosis. Further research in our laboratory will use other animal models of depression, including chronic restraint stress (CRS), learned helplessness models, and CSDS to confirm the universality of the findings reported here.

Our study first reveals that pyroptosis of astrocytes is induced during the pathogenesis of the CMS model and demonstrates an unexpectedly pivotal role of NLRP3/Casp-1/GSDMD–mediated pyroptosis in CMS model–induced behavioral changes, as well as astrocytic loss in the hippocampus. By extension, we propose that targeting the pyroptotic pathway may provide potential therapeutic benefits to the stress-related astrocytic loss in the depression.

## Methods

### Animals

All Male C57BL/6J WT mice (20 ± 2 g) used in this study were obtained from the Experimental Animal Center of Nanjing Medical University (Nanjing, China). NLRP3-cKO mice and their littermate control mice (GFAP-Cre mice and NLRP3^fl/fl^ mice), as well as Casp-1^–/–^ mice with C57BL/6J genetic background, were all obtained from Nanjing University Laboratory Animal Center (Jiangsu, China). GSDMD^–/–^ mice were shared by Shao Feng (Beijing, China). Mice were housed within standard feeding conditions (temperature 25°C ± 2°C; light/dark cycle for 12/12 hours) and given ad libitum access to food and water in the Experimental Animal Center of Nanjing Medical University.

### CMS procedures

The CMS model was widely used to explore depression-like behaviors in mice as previously described (52). Mice were singly caged and habituated to a 1% sucrose solution; then, mice were stimulated with the CMS protocol (Table 1). The protocol included a series of mildly stimulating applications, including application of cold water (4°C for 5 minutes), wet bedding (12 hours), the reversal of the light-dark cycle (24 hours), empty cage (12 hours), restraint (6 hours), food and water deprivation (24 hours), the clipping of tails (3 minutes), use of a strobe light (12 hours) and cage tilting (45°C, 12 hours). We randomly scheduled 2–3 types of stimulation daily, and they could not be repeated for 3 consecutive days. The sucrose preference of each mouse was measured weekly.

### Pharmacological treatments in mice

C57BL/6J WT mice were randomly divided into CMS, CMS + FLX, and CMS + CIT groups. FLX (F132, MilliporeSigma) and CIT (C7861, MilliporeSigma) was dissolved in 0.9% saline and i.p. injected into the experimental mice with a dose of 10 mg/kg/day. Throughout the SSRI treatment period, mice from the CMS + FLX group and CMS + CIT group were maintained on CMS stimulation. Behavioral testing commenced after 4 weeks of SSRIs treatment. Twenty-four hours after the final behavioral testing, mice were sacrificed via cardiac perfusion or cervical dislocation. Hippocampal tissue samples were frozen at –80°C or fixed in 4% paraformaldehyde for further analysis.

### Behavioral tests

#### SPT.

The SPT was used to determine the anhedonia state of animals. First, mice were habituated to a 1% sucrose solution for 48 hours before the test day. After 12 hours of food and water deprivation, mice were offered 2 bottles containing either tap water or 1% sucrose solution over the next 8 hours. To avoid side preference, we randomly alternated the position of each drinking tube every 4 hours. Water and sucrose solution consumption was calculated by measuring the change in the weight of fluid consumed. The sucrose preference was calculated from the following formula: sucrose preference (%) = sucrose intake (g)/(sucrose intake [g] + water intake [g]) × 100%.

#### FST.

The FST was used to assess depression-like behaviors in mice. Mice were individually placed in an open cylindrical container (diameter, 25 cm; height, 25 cm) containing 15 cm of water at 20°C ± 1°C for 6 minutes. The immobility time was recorded during the last 4 minutes. The mouse was considered immobile when floating passively, only with minimal movements to keep head above water. The average latency time for each mouse was calculated and recorded by Intelligent Systems Inc.

#### TST.

The TST was used to measure depression or behavioral despair in mice. The mice were suspended 50 cm above the floor by the tail via an adhesive tape at approximately 1 cm from the tip of the tail. The duration of immobility over a single 6-minute session was recorded for each mouse. Immobility was defined as the absence of any limb or body movements except those caused by respiration. The duration of immobility was recorded during the last 4 minutes by TailSuspScan.

#### SI.

The SI was performed to assess the depression-related phenotype of mice. Before the test, C57BL/6J mice were placed in the empty box (50 × 50 × 60 cm) for 5 minutes to acclimatize the environment. Then, a stimulus CD1 mouse was introduced into the wire cage in one side compartment, and an empty cage was placed in the opposite side compartment. The latency to sniff and total time spent sniffing of C57BL/6J mice were automatically measured over the course of 10 minutes by Intelligent Systems Inc. The social preference index was calculated as the time spent in SI minus the time spent exploring the empty cage (EC) divided by the total time (T) spent exploring the cages ((SI-EC)/T). Notably, exploration was defined as initiatively facing, sniffing, or touching the object (within 2 cm from the object).

#### NSF.

The NSF was used to assess anxiety-like behaviors in mice. Before the test, mice were deprived of water and food for 24 hours. In the NSF, a high-fat chow was placed in the middle of the 50 × 50 cm arena; then, mice were placed into one side of the arena. The mice were videotaped for 5 minutes, and the latency time to the first eat was automatically recorded by Intelligent Systems Inc.

#### OFT.

The OFT was used to assess the motor performance and anxiety-like behaviors of mice in an open field. In the OFT, each animal was placed in an empty arena (50 × 50 × 60 cm) in standard room lighting conditions (269 lux illumination). This arena was subdivided into 2 target regions: the edge region of the arena and the center region of the arena (35 × 35 cm). The total track length of the subject mouse in the center region of the arena was video recorded by Intelligent Systems Inc. during a 5-minute observation period.

#### PI labeling and tissue preparation.

PI (P4170, MilliporeSigma, 1 mg/mL dissolved in saline) was injected into bilateral ventricles of mice. By 2 hours after PI injection, mice were anesthetized by an i.p. injection of 10% chloral hydrate, and hippocampal tissue samples were frozen at –80°C or fixed in 4% paraformaldehyde for further analysis.

#### Injection of AAV.

Microinjectors (Genechem) were bilaterally implanted into the ventral hippocampus (AP = –2.3 mm, ML = ± 1.6 mm, DV = + 1.8 mm) of each mouse under the aseptic condition. The serotype of AAV is AAV-9. Then, AAV-GFAP-EGFP or AAV-GFAP-mGSDMD-EGFP was performed bilaterally into the ventral hippocampus at the rate of 0.2 μL/min (1 μL/side) for 1 minute. After the injection, the mice were allowed to recover at room temperature (RT). After a 4-week recovery, mice were subjected to 6-week CMS stimulation.

#### Immunofluorescence analysis.

Brain sections were performed as previously reported (53) and immunostained with the following primary antibodies: mouse anti–Casp-1 monoclonal antibody (1:200, AG-20B-0042, AdipoGen), anti-GFAP polyclonal antibody (1:1000, MAB360, MilliporeSigma), rabbit anti-MLKL (phospho S345) polyclonal antibody (1:100, ab196436, Abcam), rabbit anti–Casp-3 polyclonal antibody (1:200, 9662, Cell Signaling Technology [CST]), rabbit anti–Iba-1 polyclonal antibody (1:1000, 019-19741, Wako), and rabbit anti-NeuN polyclonal antibody (1:200, ab177487, Abcam). The secondary antibodies used were as follows: Alexa Fluor 555 goat anti–mouse IgG (1:1000, A-21422, Invitrogen) and Alexa Fluor 488 goat anti–rabbit IgG (1:1000, A-11001, Invitrogen). Nucleus were stained with Hoechst (1:1000 diluted in PBS, B2883, MilliporeSigma). Fluorescence images were captured by a confocal laser-scanning microscope (Leica).

#### TSA coupled multiplex fluorescence staining.

Brain slices were treated in 0.03% H_2_O_2_ in 0.01M PBS for 15 minutes. After washing, slices were treated at 4°C for 24 hours with a mouse antibody against the GFAP (1:1000, MAB360, MilliporeSigma) diluted in PBS containing 0.5% BSA and 0.25% Triton X-100. Slices were then treated with a swine anti-mouse/horseradish peroxidase conjugate (Thermo Fisher Scientific) diluted 1:1000 in TNB buffer (0.1M Tris-HCl, pH 7.5; 0.15M NaCl; 0.05% Tween 20; MilliporeSigma) for 1 hour. Sections were washed 3 times in 0.01M PBS and incubated in a biosignal tyramide-fluorescein (BT-FITC) conjugate (*NEN* Life Sciences Products) diluted 1:100 in amplification diluent for 20 seconds at RT. Sections were washed for 3 times, for 5 minutes each time, in sterilized distilled water and then were cover-slipped with 2.5% 1,4-diazabicyclo[2.2.2]octane (DABCO; MilliporeSigma) in glycerol (MilliporeSigma) for 1 hour at RT. After rinsing, sections were treated at 4°C for 24 hours with a mouse antibody against the Casp-1 (1:1000, AG-20B-0042, AdipoGen) diluted in PBS containing 0.5% BSA and 0.25% Triton X-100. The following steps were the same as above. For the last step, sections were stained with Hoechst (B2883, MilliporeSigma) for 10 minutes in the dark place at RT. Images were captured by confocal laser-scanning microscope (Leica).

### Western blotting analysis

Proteins were separated within 10%–15% SDS-PAGE and then transferred to the PVDF membrane and treated with blocking buffer at RT for 1 hour. PVDF membranes were then treated with antibodies as follows: mouse anti-NLRP3 monoclonal antibody (1:1000, AG-20B-0014-C100, AdipoGen), mouse anti–Casp-1 monoclonal antibody (1:1000, AG-20B-0042, AdipoGen), rabbit anti–Casp-11 polyclonal antibody (1:1000, 14340, CST), rabbit anti-GSDMD polyclonal antibody (1:500, G7422, MilliporeSigma), goat anti–IL-1β polyclonal antibody (1:1000, I3767, MilliporeSigma), and mouse anti–β-actin monoclonal antibody (1:1000, sc-47778, Santa Cruz Biotechnology Inc.) overnight at 4°C. Then membranes were washed and treated with HRP-conjugated secondary antibodies: HRP-conjugated affinipure goat anti–rabbit IgG (H+L) (1:5000, SA00001-2, Proteintech) and HRP-conjugated affinipure goat anti-mouse IgG (H+L) (1:5000, SA00001-1, Proteintech). An electrochemiluminescence (ECL) Western blotting assay system (Pierce Biotechnology) was used to evaluate protein expression levels for protein expression analysis.

### Statistics

Values were represented as the mean ± SEM. Data were analyzed using 2-tailed Student’s *t* test, 1-way ANOVA, 2-way ANOVA, or repeated-measures ANOVA. They were then combined with post hoc Tukey-Kramer test to assess the differences between groups. *P* < 0.05 was regarded as significant. All statistical analyses were implemented by Graphpad Prism (version 8.0).

### Study approval

All animal procedures were performed according to the approved protocol by the IACUC of Nanjing Medical University (no. 1903038). Animal handling and laboratory procedures were based on the NIH guidelines for animal research.

## Author contributions

SL and Y Sun carried out the majority of the experimental work, analyzed data, and helped to draft the manuscript. MS, Y Song, YF, and QZ contributed to the experimental work. XL, NS, and JD advised on and helped carry out and analyze the behavioral tasks. ML took charge of paper writing, designing, and performing the experiments. GH was primarily responsible for making experiment plans.

## Supplementary Material

Supplemental data

## Figures and Tables

**Table 1:**
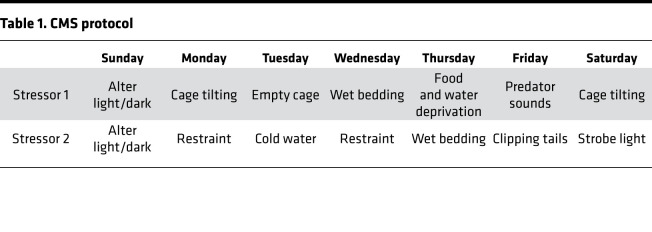
CMS protocol

## Version 1 12/08/2021

Electronic publication
