# Supplementary material for: NLRP3/caspase-1/GSDMD–mediated pyroptosis exerts a crucial role in astrocyte pathological injury in mouse model of depression
Source: JCI Insight. 2021 Dec 8;6(23):e146852. doi: 10.1172/jci.insight.146852 (PMC8675200; doi:10.1172/jci.insight.146852)
Supplement: Supplemental data [file jciinsight-6-146852-s189.pdf]

Supplementary Figures

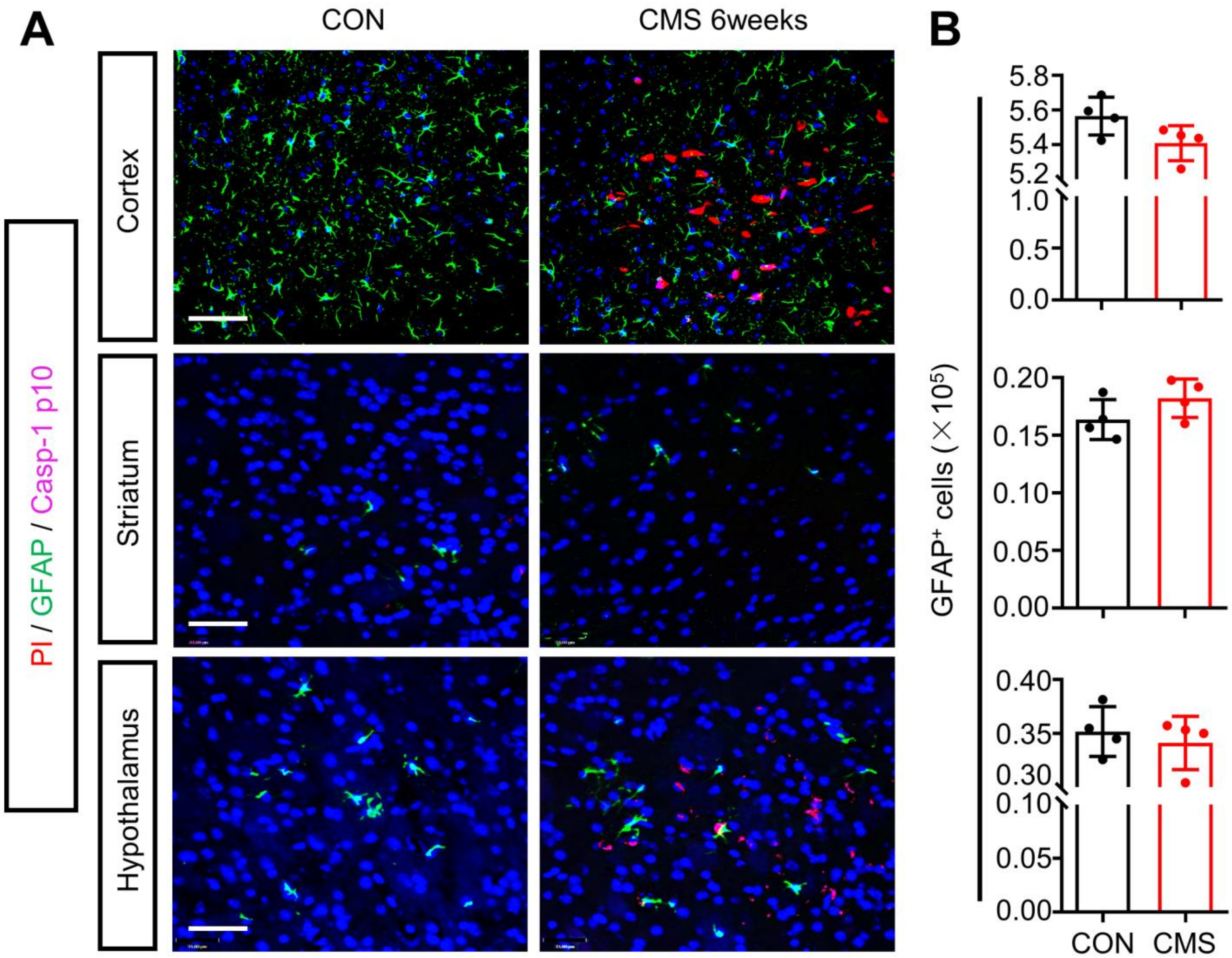

Figure S1. related to Figure 1, Astrocytic pyroptosis in different brain regions of mice. CMS model was performed for 6 weeks. **(A)** GFAP (green), Casp-1 p10 (magenta), PI (red)-labeled cells in a portion of the ipsilateral cortex, striatum and hypothalamus from one animal injected with vehicle or 1 μL of PI following CMS stimulus by TSA coupled multiplex fluorescent staining. Scale bar = 50 μm. **(B)** Densitometric analysis of numbers of GFAP-positive cells in the DG region of hippocampus. *n* = 4 mice per group. Values were represented as mean ± SEM. Data were analyzed using Student's *t*-test.

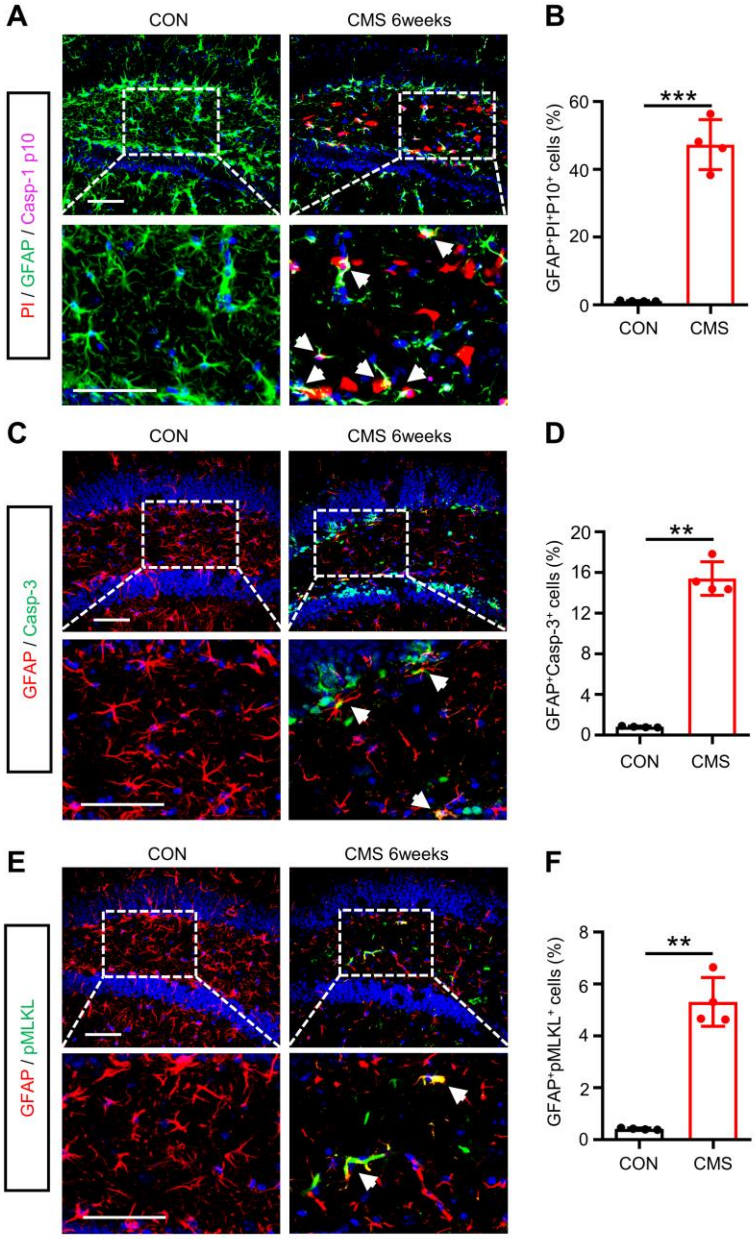

Figure S2. related to Figure 1, Levels of apoptosis and necroptosis in astrocytes in the hippocampus of mice. CMS model was performed for 6 weeks. **(A)** GFAP (green), Casp-1 p10 (magenta), PI (red)-labeled cells in a portion of the ipsilateral ipsilateral DG hippocampal region from one animal injected with vehicle or 1 μL of PI following CMS stimulus by TSA coupled multiplex fluorescent staining. Scale bar = 50 μm. **(B)** Densitometric analysis of numbers of GFAP-positive cells in the DG region of hippocampus. *n* = 4 mice per group. **(C-D)** The expression of Casp-3 on astrocytes in the hippocampal DG region. White arrowheads show the examples of Casp-3 and GFAP double positive cells. **(E-F)** The expressions of pMLKL on astrocytes in the hippocampal DG region. White arrowheads show the examples of pMLKL and GFAP double positive cells. Scale bar = 50 μm. *n* = 4 mice per group. Values were represented as mean ± SEM. Data were analyzed using Student's *t*-test. $^{**}P < 0.01$, $^{***}P < 0.001$.

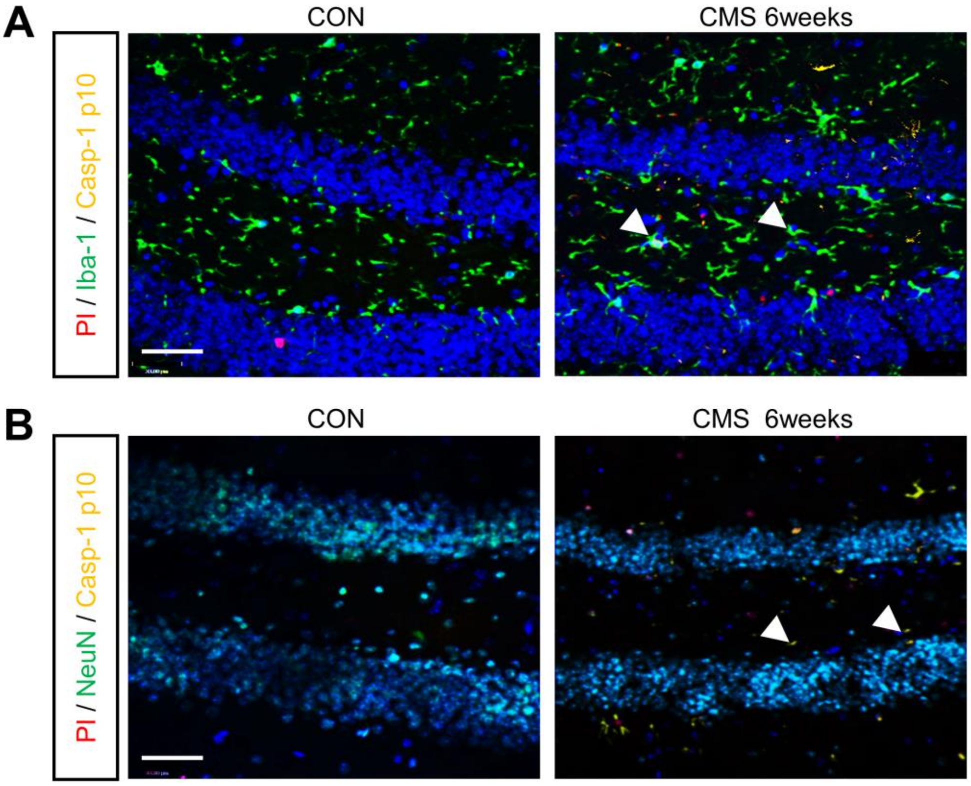

Figure S3. related to Figure 1, No microglial or neuronal pyroptosis was seen in the hippocampus of CMS mice. CMS model was performed for 6 weeks. **(A)** Iba-1 (green), Casp-1 p10 (yellow), PI (red)-labeled cells in a portion of the ipsilateral DG hippocampal region from one animal injected with vehicle or 1 μL of PI following CMS stimulation by TSA coupled multiplex fluorescent staining. **(B)** NeuN (green), Casp-1 p10 (yellow), PI (red)-labeled cells in a portion of the ipsilateral DG hippocampal region from one animal injected with vehicle or 1 μL of PI following CMS stimulus by TSA coupled multiplex fluorescent staining. Scale bar = 50 μm. *n* = 4 mice per group.

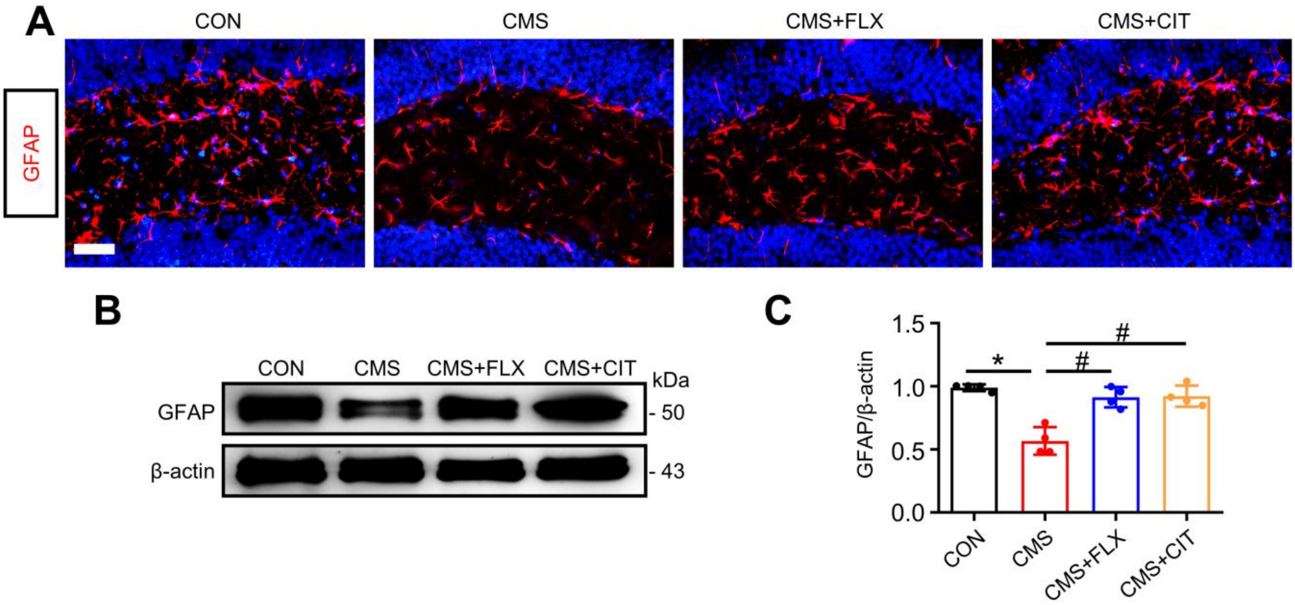

Figure S4. related to Figure 2, SSRIs improved astrocyte loss in the hippocampus of CMS mice. CMS was performed for 6 weeks. C57BL/6 mice were then injected consecutively with FLX (10 mg/kg) or CIT (10 mg/kg) by daily i.p for 4 weeks. **(A)** Astrocytes were analyzed by immunostaining. Red color represents GFAP and blue color represents nucleus. Scale bar = 50 μm, photos were taken at ×20 magnification. **(B)** The protein level of hippocampal GFAP was analyzed by immunoblotting. **(C)** Densitometric analysis of GFAP. n = 4 mice per group. Values were represented as mean ± SEM. Data were analyzed using one-way ANOVA，then combined with unpaired t-test to assess the differences between groups. $^{*}P < 0.05$ *vs.* CON group, $^{\#}P < 0.05$ *vs*. CMS group. i.p, intraperitoneal injection; CON, Control; CMS, Chronic mild stress; FLX, Fluoxetine; CIT, Citalopram.

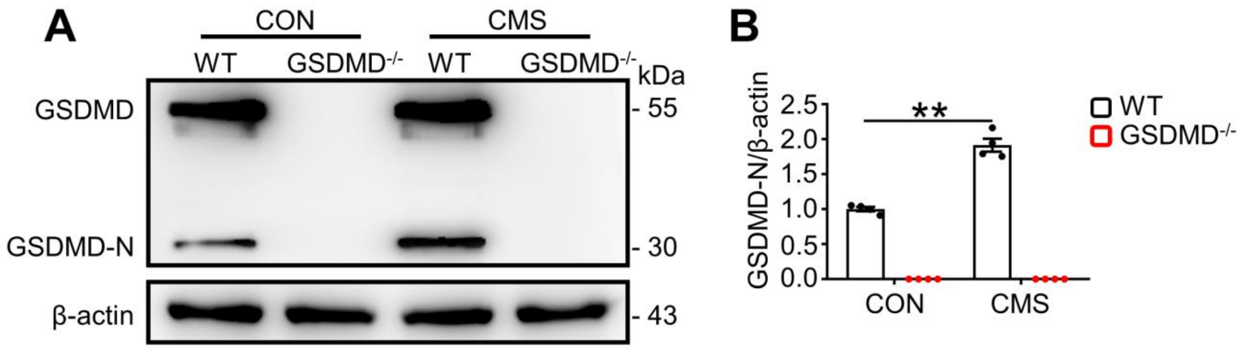

Figure S5. related to Figure 3, Validation of gene knockout efficiency in the hippocampus of GSDMD knockout mice. (A) Immunoblotting was used to analyze the expression of GSDMD and GSDMD-N from mice hippocampal homogenate. **(B)** Densitometric analysis of GSDMD-N. $n = 4$ mice per group. Values were represented as means ± SEM. Data were analyzed using two-way ANOVA, then combined with Tukey to assess the differences between groups. $^{**}P < 0.01$.

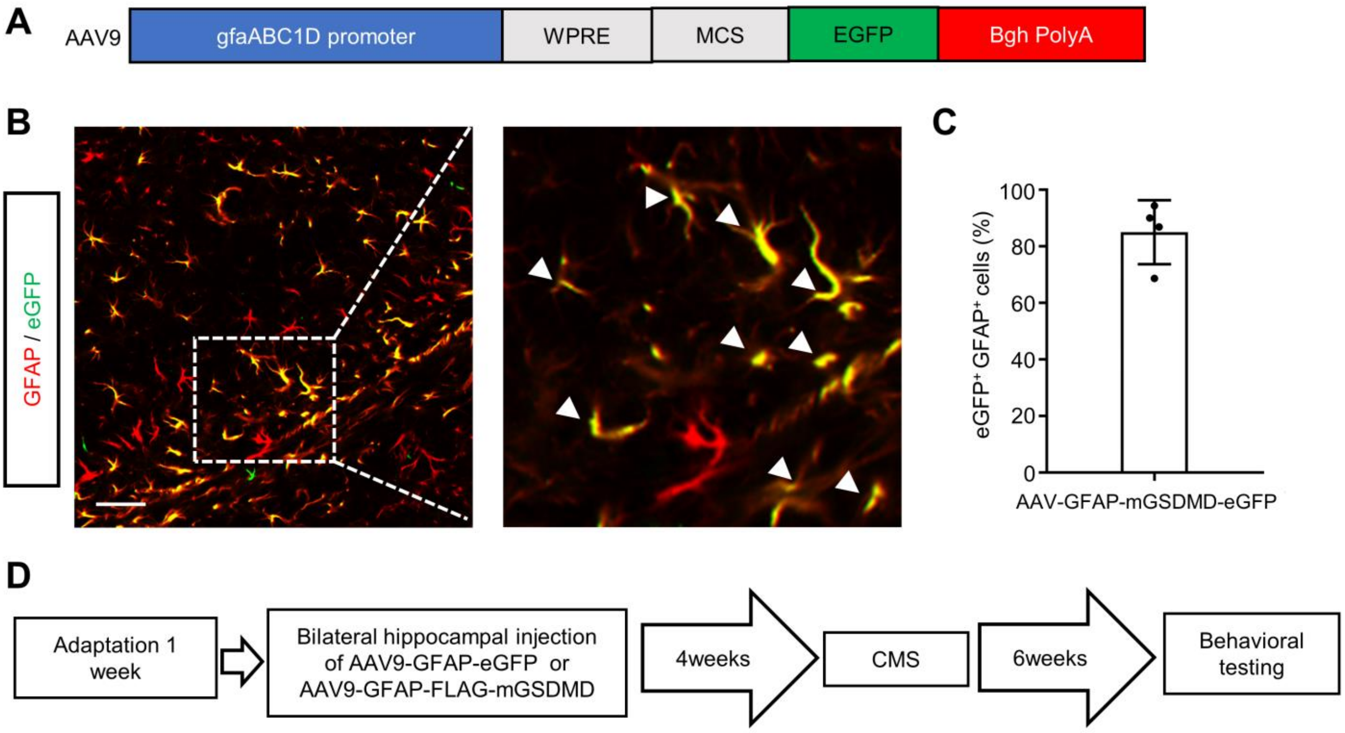

Figure S6. related to Figure 4, The experimental flow and infection efficiency of overexpression of GSDMD-N gene fragments in astrocytes. **(A)** Schematic representation of construct showing mouse GSDMD-N subcloned into an AAV9 plasmid under transcriptional regulation of the GFAP promoter (AAV9-gfaABC1D promoter-WPRE-MCS-EGFP-Bgh Poly). AAV9-gfaABC1D promoter-WPRE-MCS-EGFP-Bgh PolyA plasmid without encoding GSDMD served as the control. **(B)** Representative photomicrographs of injection sites in the hippocampus. Scale bar = 200 μm. **(C)** The infection efficiency of overexpression of GSDMD-N gene fragments in astrocytes. *n* = 4 mice per group. **(D)** The timeline of experimental procedure for mice.

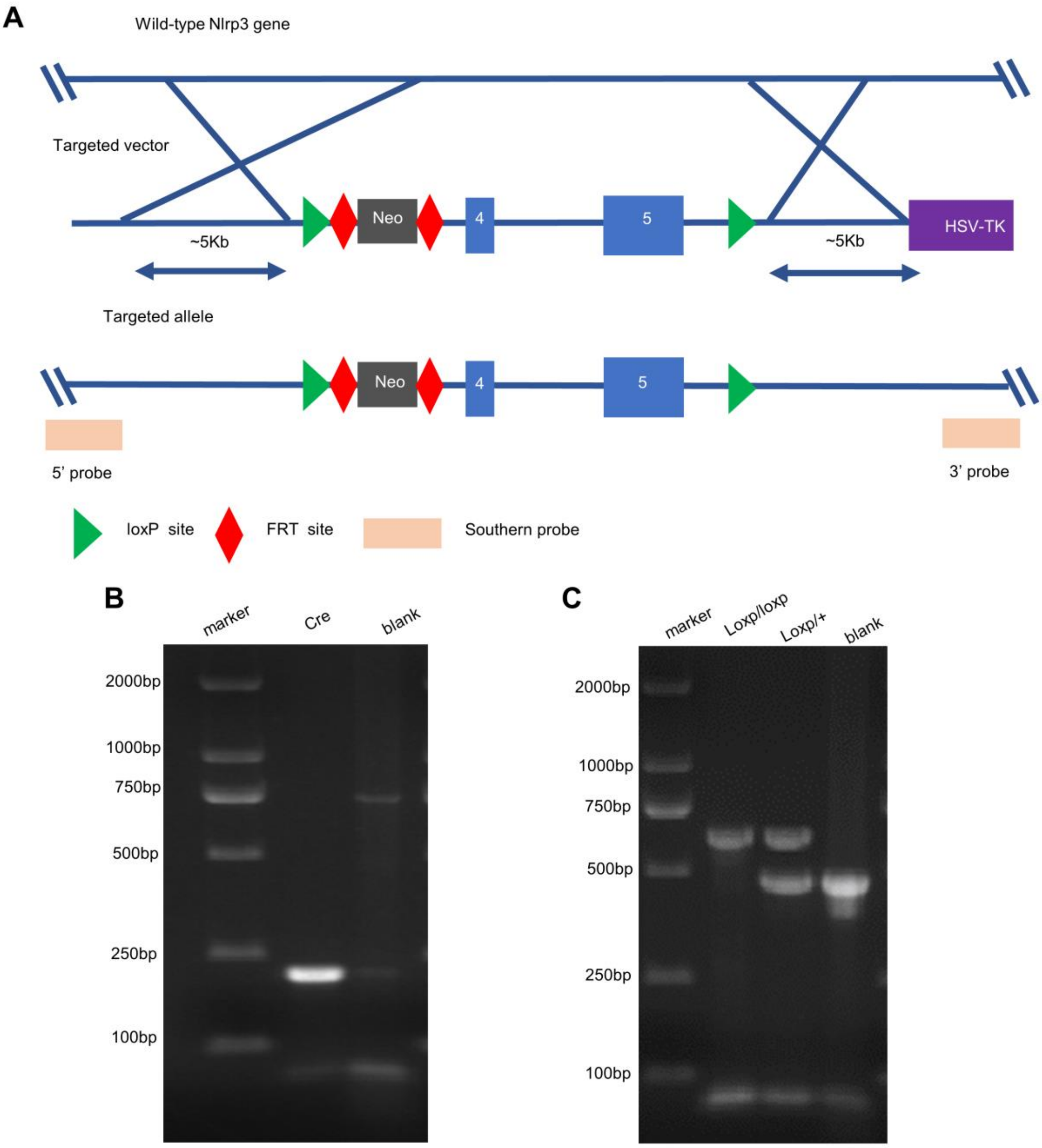

Figure S7. related to Figure 6, Strategy of transgenic mice with selective deficiency and DNA identification results of Nlrp3 gene in astrocytes. **(A)** Schematic drawing of floxed NLRP3 gene construct, which was used to generate a mouse line allowing cell-specific knockout of NLRP3 gene in astrocytes. **(B)** Genotyping of GFAP-Cre mice by PCR. **(C)** Genotyping of NLRP3$^{flox/flox}$ mice by PCR. Representative images were selected from at least 3-time repeated experiments.
